# Limonoid 7-Deacetoxy-7-oxogedunin (CG-1) Attenuates RANKL-Induced Osteoclastogenesis via Inhibiting PI3K/Akt-NFATc1 Axis

**DOI:** 10.3390/cells15100854

**Published:** 2026-05-07

**Authors:** Atsushi Koike, Ko Fujimori

**Affiliations:** Department of Pathobiochemistry, Faculty of Pharmacy, Osaka Medical and Pharmaceutical University, 4-20-1 Nasahara, Takatsuki 569-1094, Osaka, Japan; atsushi.koike@ompu.ac.jp

**Keywords:** limonoid, 7-deacetoxy-7-oxogedunin (CG-1), RANKL, osteoclastogenesis, Akt, NFATc1

## Abstract

**Highlights:**

**What are the main findings?**

- CG-1 attenuates RANKL-induced activation of the PI3K/Akt–NFATc1 axis.
- CG-1 interferes with an early osteoclastogenic signaling cascade.

**What are the implications of the main findings?**

- CG-1-inhibited RANKL-induced osteoclastogenesis.
- CG-1 has the potential to inhibit the progression of osteoclast-related bone diseases.

**Abstract:**

Excessive bone resorption by osteoclasts causes pathological bone loss in diseases such as osteoporosis. 7-Deacetoxy-7-oxogedunin (CG-1), a limonoid isolated from *Carapa guianensis* (Meliaceae), exhibits various biological activities. Here, we examined the anti-osteoclastogenic effect of CG-1 and its underlying mechanism in receptor activator of NF-κB ligand (RANKL)-induced osteoclast differentiation of RAW264.7 cells. CG-1 inhibited the formation of tartrate-resistant acid phosphatase-positive multinucleated cells and decreased the expression of osteoclastogenesis-related genes. When CG-1 was added to the culture during the first 3 days of the 5-day-osteoclastogenesis period, the expression levels of the osteoclastogenesis-related genes (*Nfatc1*, *Acp5*, *Src*, *Ctsk*, and *Mmp9*) were decreased, as was observed when CG-1 was added continuously for 5 days. Furthermore, CG-1 lowered RANKL-induced Akt phosphorylation, which is similar to the results seen with the PI3K inhibitor, LY294002. Moreover, CG-1 and LY294002 suppressed the RANKL-induced expression of NFATc1, the master transcription factor for regulating terminal differentiation into osteoclasts. These results suggest that CG-1 attenuated RANKL-induced osteoclastogenesis by inhibiting the PI3K/Akt-NFATc1 axis during the early stage of osteoclast differentiation. Thus, CG-1 has the potential to suppress osteoclast-mediated bone resorption.

## 1. Introduction

Bone remodeling is a lifelong process that is tightly regulated by the cooperative activities of bone-forming osteoblasts and bone-resorbing osteoclasts. The balance between bone formation and bone resorption is critical for maintaining skeletal integrity, while facilitating the repair of microdamage and regulating mineral metabolism [1]. However, disruption of this equilibrium, primarily due to excessive osteoclast activation, causes bone diseases such as osteoporosis and rheumatoid arthritis, which are characterized by bone loss and compromised microarchitecture. These diseases have now become a global health concern [2]. The current drugs used for the treatment of these diseases include bisphosphonates, selective estrogen receptor modulators, and monoclonal antibodies such as denosumab [3,4].

Osteoclasts are large multinucleated cells that are differentiated from the monocyte/macrophage hematopoietic lineage [5]. Differentiation, activation, and survival of osteoclasts are primarily regulated by two cytokines, namely, macrophage colony-stimulating factor (M-CSF) and receptor activator of nuclear factor-κB (NF-κB) ligand (RANKL) [5,6]. M-CSF promotes the proliferation and survival of osteoclast precursor cells, whereas RANKL drives differentiation from osteoclast precursor cells into mature osteoclasts [7,8]. The binding of RANKL to its cognate receptor, RANK, on osteoclast precursor cells activates the intracellular signaling cascades, including the phosphoinositide 3-kinase (PI3K)/protein kinase B (Akt) signaling pathway [9]. This signaling pathway activates nuclear factor of activated T-cells cytoplasmic 1 (NFATc1) [10,11] that is the master transcription factor of osteoclast differentiation and enhances the expression of osteoclastogenesis-related genes including acid phosphatase 5 [*Acp5*, also called tartrate-resistant acid phosphatase (*Trap*)], proto-oncogene tyrosine-protein kinase Src (*Src*), cathepsin K (*Ctsk*), and matrix metallopeptidase 9 (*Mmp9*) [12]. Therefore, the RANKL-activated signaling pathway in osteoclastogenesis is a viable target for the development of anti-resorptive drugs. Denosumab, a human monoclonal antibody to RANKL, has been developed as an anti-resorptive drug for treating osteoporosis. Bisphosphonates, another class of anti-resorptive drugs, are analogs of inorganic pyrophosphate that strongly bind to bone and reduce bone resorption. These drugs are the first-line therapy for osteoporosis [13]; however, they often cause adverse effects such as osteonecrosis of the jaw and atypical femoral fractures [14].

Natural products have beneficial effects in regulating various biological functions, including antioxidant, anti-tumor, and anti-bone resorption activities [15]. Natural products generally exhibit moderate effects and few adverse effects [16]. Limonoids are tetracyclic triterpenoids with diverse biological activities [17]. Nomilin, a limonoid found in citrus fruits, inhibits osteoclastogenesis by suppressing the NFATc1 and mitogen-activated protein kinase (MAPK) signaling pathways [18].

7-Deacetoxy-7-oxogedunin (CG-1), a limonoid, is present in the seeds of the medicinal plant *Carapa guianensis* (Meliaceae), commonly known as andiroba [19]. CG-1 exhibits anti-lipogenic and anti-obesity activities [20,21,22]. Additionally, CG-1 inhibits RANKL-induced osteoclastogenesis by suppressing the NF-κB and MAPK signaling pathways in the very early stage after RANKL stimulation [23]. However, the critical regulatory mechanism including the downstream regulation by CG-1-induced anti-osteoclastogenic effect remains unclear. In the present study, we examined the stage-specific effect of CG-1 and whether the PI3K/Akt-NFATc1 axis is involved in its inhibitory action during RANKL-induced osteoclast differentiation.

## 2. Materials and Methods

### 2.1. Materials

CG-1 was kindly provided from Prof. Reiko Tanaka (Osaka Medical and Pharmaceutical University, Japan) [24]. *N*,*N*-dimethylformamide, *p*-nitrophenyl phosphate, sodium tartrate, ferric chloride hexahydrate, sodium orthovanadate, and sodium molybdate were purchased from FUJIFILM Wako Pure Chemical (Osaka, Japan). Sodium fluoride was purchased from Nacalai Tesque (Kyoto, Japan). Naphthol AS-MX phosphate, Fast Red Violet LB salt, and dimethyl sulfoxide (DMSO) were obtained from Sigma-Aldrich (St. Louis, MO, USA). LY294002 was purchased from Selleck Chemicals (Houston, TX, USA). Primary antibodies against phospho-Akt (#4060; 1:1000), Akt (#9272; 1:1000), phospho-p38 (#9216; 1:1000), p38 (#9212; 1:1000), phospho-ERK (#9101; 1:1000), ERK (#9102; 1:1000), phospho-JNK (#9251; 1:1000), JNK (#9252; 1:1000), and IκB (#9242; 1:1000) and secondary anti-rabbit IgG (#7074; 1:1000) and anti-mouse IgG (#7076; 1:1000) HRP-linked antibodies were obtained from Cell Signaling Technology (Danvers, MA, USA). Purified anti-NFATc1 antibody (#649601; 1:500) was purchased from Biolegend (San Diego, CA, USA). Anti-GAPDH antibody (60004-Ig; 1:5000) was obtained from Proteintech (Rosemont, IL, USA). Recombinant human soluble RANKL (sRANKL) was obtained from Oriental Yeast (Tokyo, Japan). Preparation of glutathione S-transferase (GST) and GST-RANKL proteins is described in Supporting Information [25].

### 2.2. Cell Culture

The mouse macrophage-like cell line RAW264.7 was purchased from American Type Culture Collection (ATCC, Manassas, VA, USA). The cells were cultured in Dulbecco’s Modified Eagle’s Medium (DMEM; FUJIFILM Wako Pure Chemical) containing 10% (*v*/*v*) fetal calf serum (FCS; Thermo Fisher Scientific; Waltham, MA, USA) and Penicillin–Streptomycin Mixed Solution (PS; 50 U/mL penicillin and 50 μg/mL streptomycin; Nacalai Tesque) at 37 °C in a humidified atmosphere containing 5% CO_2_.

### 2.3. Cell Viability Assay

RAW264.7 cells were seeded at a density of 5 × 10^3^ cells/well in 96-well plates and cultured for 24 h in phenol red-free Minimum Essential Medium (MEM; Thermo Fisher Scientific) containing 10% (*v*/*v*) FCS, PS, and MEM Non-Essential Amino Acids Solution (NEAA; Nacalai Tesque). The medium was discarded and changed to the same fresh medium, and the cells were cultured further. Subsequently, the medium was replaced on day 3, and the cell culture was continued for 2 more days. CG-1 (0–20 μM) was added when the medium was changed. Cell viability was assessed using a Cell Counting Kit-8 (Dojindo; Kumamoto, Japan) following instructions supplied by the manufacturer.

### 2.4. Osteoclast Differentiation

RAW264.7 cells were seeded at a density of 5 × 10^4^ cells/well in 12-well plates in MEM containing 10% (*v*/*v*) FCS, PS, and NEAA. After 24 h, the medium was replaced with the same fresh medium plus each of the following treatments: (i) 50 ng/mL of GST alone as a negative control; (ii) 50 ng/mL of GST-RANKL with vehicle [0.1% (*v*/*v*) DMSO] as the differentiation control; or (iii) 50 ng/mL of GST-RANKL with CG-1 at final concentrations of 1, 3, or 5 µM. The culture medium for each treatment was changed to the same fresh medium on day 3, and the cells were cultured for further 2 days to allow for differentiation into osteoclasts.

### 2.5. TRAP Staining Assay

The TRAP staining solution was freshly prepared as follows. Five mg of Naphthol AS-MX phosphate was dissolved in 500 µL of *N*,*N*-dimethylformamide. Fast Red Violet LB salt (30 mg) was dissolved in 50 mL of 0.1 M sodium acetate buffer (pH 5.0) containing 50 mM sodium tartrate. The two solutions were then mixed to prepare the TRAP staining solution.

RAW264.7 cells were seeded at a density of 5 × 10^4^ cells/well in 12-well plates and cultured in MEM supplemented with 10% (*v*/*v*) FCS, PS, and NEAA with GST (50 ng/mL) or GST-RANKL (50 ng/mL) in the presence of CG-1 (0–5 μM) or not. On day 3, the medium was discarded and replaced with the same fresh medium, and the cell culture was continued for 2 more days. The cells were washed with PBS(−) twice and fixed with 4% (*w*/*v*) paraformaldehyde at 25 °C for 10 min. The cells were permeabilized by rinsing briefly with ethanol:acetone (1:1, *v*/*v*) and then allowing them to air dry. Then, the cells were incubated with the TRAP staining solution at 37 °C for 1 h. After washing the cells with PBS(−), TRAP-positive, purple-red cells containing three or more nuclei were identified as mature osteoclasts under a microscope (CKX41; Olympus; Tokyo, Japan).

### 2.6. TRAP Activity

RAW264.7 cells were seeded at a density of 5000 cells/well in 96-well plates and differentiated into osteoclasts in MEM containing 10% (*v*/*v*) FCS, PS, and NEAA with GST (50 ng/mL) or GST-RANKL (50 ng/mL) in the presence of CG-1 (0–5 μM) or not. The medium was discarded and changed to the same fresh medium on day 3, after which the cells continued to culture for 2 additional days. After 5 days, the cells were washed with PBS(−), and lysed in 100 µL of lysis buffer containing 0.1% (*v*/*v*) Triton X-100 in PBS(−) on ice for 10 min.

For the TRAP assay, a 10 µL aliquot of the resulting cell lysates was transferred to 96-well plates. The enzymatic reaction began with the addition of 100 µL of substrate solution including 2.5 mM *p*-nitrophenyl phosphate dissolved in TRAP reaction buffer [0.1 M sodium acetate (pH 5.0), 50 mM sodium tartrate, 0.1% (*v*/*v*) Triton X-100, 1 mM sodium ascorbate, and 1 mM ferric chloride hexahydrate] at 37 °C for 1 h. The reaction was terminated by adding 50 µL of 0.9 M NaOH. We measured the amount of *p*-nitrophenol at 405 nm using a MultiSkan FC microplate reader (Thermo Fisher Scientific).

### 2.7. Quantitative Real-Time PCR (qRT-PCR)

Total RNA was extracted using RNAiso Plus (Takara-Bio; Shiga, Japan). Total RNA (One µg) was utilized to synthesize the cDNAs by use of ReverTra Ace qPCR RT Master Mix (Toyobo; Osaka, Japan) following the manufacturer’s instructions. The resulting cDNAs were used as the templates for qRT-PCR on a LightCycler 96 System (Roche Diagnostics; Mannheim, Germany) using TB Green Premix Ex Taq (Takara-Bio). The relative gene expression level was calculated using the 2^−ΔΔCT^ method [26] and normalized to the internal reference gene, TATA-box binding protein (*Tbp*). The nucleotide sequences of primers used for qRT-PCR were as follows: *Acp5* forward, 5′-GACGATGGGCGCTGACTTCA-3′ and reverse, 5′-CGCTTGGAGATCTTAGAGT-3′; *Src* forward, 5′-TGAGCCAGGATCTGAACCA-3′ and reverse, 5′-TCCTGCTCCGTGTCCCTA-3′; *Ctsk* forward, 5′-CTGAAGATGCTTTCCCATATGTGGG-3′ and reverse, 5′-GCAGGCGTTGTTCTTATTCCGAGC-3′; *Mmp9* forward, 5′-CGAGTGGACGCGACCGTAGTTGG-3′ and reverse, 5′-CAGGCTTAGAGCCACGACCATGCAG-3′; *Tbp* forward, 5′-GGGGAGCTGTGATGTGAAGT-3′ and reverse, 5′-GGCGGTTTGGCTAGGTTT-3′.

### 2.8. Western Blot Analysis

The cells were lysed in RIPA lysis buffer including 1% (*v*/*v*) Protease Inhibitor Cocktail (Nacalai Tesque), 1 mM sodium orthovanadate, 5 mM sodium molybdate, and 5 mM sodium fluoride. Protein concentrations were measured by using a Protein Assay BCA Kit (Nacalai Tesque) following the manufacturer’s instructions. Proteins (10–20 µg) were resolved on 5–20% SDS-PAGE gels (ATTO; Tokyo, Japan) and transferred onto the polyvinylidene fluoride membranes (Immobilon-P; Merck Millipore, Burlington, MA, USA). To prevent non-specific binding, the membranes were incubated with Blocking One (Nacalai Tesque) at 25 °C for 1 h. The membranes were subsequently probed overnight at 4 °C with the appropriate primary antibody, followed by incubation with the respective horseradish peroxidase (HRP)-conjugated secondary antibody at 25 °C for 1 h. The protein bands were visualized using an ECL Prime Western Blotting Detection Reagent (Cytiva; Marlborough, MA, USA) and an iBright CL1500 Imaging System (Thermo Fisher Scientific). Band intensity was quantified using ImageJ software (Fiji version 2.9.0) [27], and each signal was normalized to glyceraldehyde-3-phosphate dehydrogenase (GAPDH) as the loading control.

### 2.9. Statistical Analysis

All data were presented as the mean ± standard deviation (SD). Statistical comparison between the two groups was performed using an unpaired Student’s *t*-test. For comparison among multiple groups, a one-way analysis of variance (ANOVA) was used, followed by a post hoc test. All statistical analyses were performed using GraphPad Prism version 10 (GraphPad Software; San Diego, CA, USA). A *p*-value of less than 0.05 was considered statistically significant.

## 3. Results

### 3.1. Expression and Purification of GST-RANKL Protein

GST-RANKL or GST protein was expressed in Escherichia coli and affinity-purified, as described in the Supporting Information. SDS-PAGE analysis showed specific bands at approximately 45 kDa, which is almost consistent with the predicted molecular weight of 44 kDa [GST: 26 kDa + RANKL extracellular domain (amino acid residues 157–316: 18 kDa)] for GST-RANKL or at 26 kDa for GST (Supplemental Figure S1). The purified GST-RANKL induced the formation of TRAP-positive multinucleated cells from RAW264.7 cells (Supplemental Figure S2). Thus, GST-RANKL activated osteoclast differentiation, which was almost comparable to that of the recombinant sRANKL (Supplemental Figure S2). However, GST alone did not induce osteoclast differentiation. Thus, GST-RANKL was used in the subsequent experiments.

### 3.2. Effect of CG-1 on the Viability of RAW264.7 Cells

**Figure 1:**
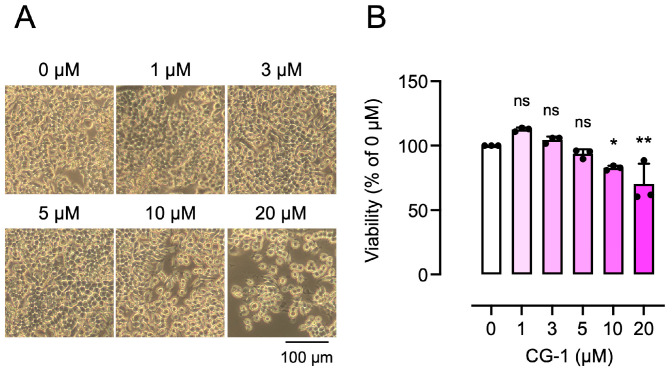
Effect of CG-1 on the viability of RAW264.7 cells. (**A**) Morphology of RAW264.7 cells treated with CG-1 (0–20 µM) for 5 days. Representative images are shown (*n* = 3). (**B**) Cell viability of RAW264.7 cells treated with CG-1 (0–20 µM) for 5 days. Data are presented as the mean ± SD of three independent experiments. ns, not significant; * *p* < 0.05, ** *p* < 0.01 vs. vehicle (0 µM).

We first evaluated the cytotoxic effect of CG-1 on RAW264.7 cells. The cells were cultured with various concentrations of CG-1 (0–20 μM) for 5 days. The cell number decreased in a CG-1 concentration-dependent manner (≥10 μM; Figure 1A). Moreover, cell viability assay confirmed that CG-1 did not affect cell viability up to 5 μM (93.7 ± 3.5%). In contrast, the cytotoxic effect of CG-1 was observed at 10 μM (82.8 ± 1.7%, *p* < 0.05) and 20 μM (70.3 ± 15.8%, *p* < 0.01) (Figure 1B). Therefore, CG-1 concentrations up to 5 μM were used in the subsequent experiments.

### 3.3. CG-1 Inhibits RANKL-Induced Osteoclastogenesis

**Figure 2:**
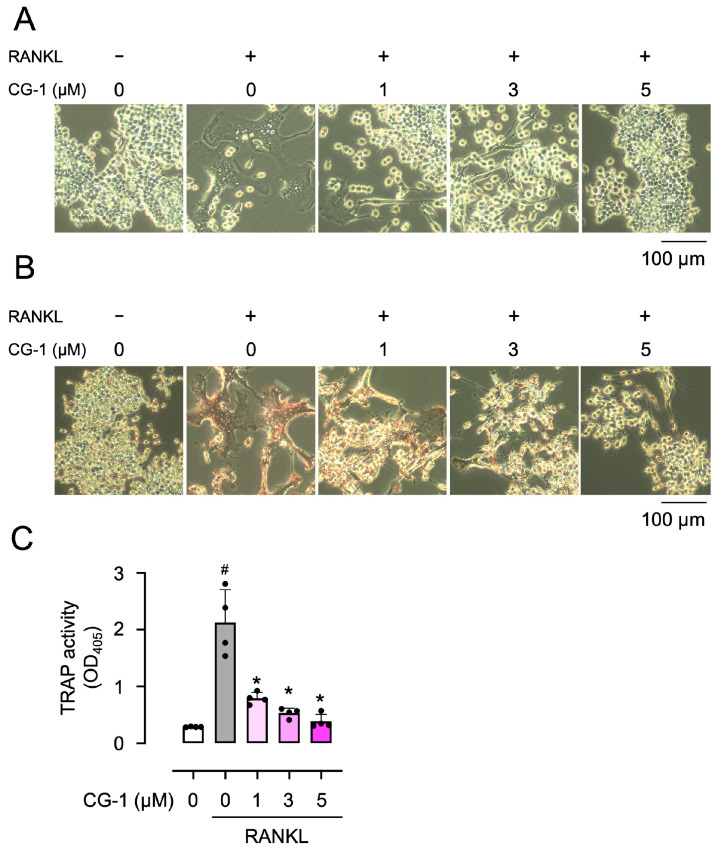
CG-1 inhibits RANKL-induced osteoclastogenesis. (**A**) Morphology of RAW264.7 cells treated with GST-RANKL (50 ng/mL) and CG-1 (0–5 µM) for 5 days. Representative images are shown (*n* = 4). (**B**) TRAP staining of RAW264.7 cells treated as stated in (**A**). Representative images are shown (*n* = 4). (**C**) TRAP activity in RAW264.7 cells treated as stated in (**A**). Data are presented as the mean ± SD of four independent experiments. # *p* < 0.01 vs. vehicle (0 μM); * *p* < 0.05 vs. RANKL alone.

RAW264.7 cells were incubated with GST-RANKL together with CG-1 (0–5 μM) to assess the effects of CG-1 on osteoclast differentiation. The number of large multinucleated cells increased when the cells were treated with GST-RANKL (Figure 2A). In contrast, GST-RANKL-induced formation of large multinucleated cells was repressed at 1 μM of CG-1 and almost completely suppressed at concentrations of ≥3 μM (Figure 2A).

Then, we performed TRAP staining to confirm osteoclast differentiation of RAW264.7 cells. Figure 2B shows that the number of TRAP-positive cells increased when the cells were cultured in the presence of GST-RANKL, compared to cells treated with GST alone. When the cells were treated with GST-RANKL together with various concentrations of CG-1 (0–5 μM), the number of TRAP-positive cells decreased concentration-dependently, compared to that seen in GST-RANKL-treated cells, consistent with the results shown in Figure 2A. Moreover, when the cells were incubated with GST-RANKL, TRAP activity was elevated by approximately 8.3-fold, compared to that of GST alone-treated cells (Figure 2C). GST-RANKL-induced TRAP activity was reduced by co-treatment with CG-1 by 66.9 ± 4.2%, 77.2 ± 3.7%, and 81.5 ± 6.9% at 1, 3, and 5 μM, respectively, of the GST-alone treatment (Figure 2C). These results reveal that CG-1 inhibited RANKL-induced osteoclast differentiation in a concentration-dependent manner.

### 3.4. CG-1 Decreases the Expression of RANKL-Induced Osteoclastogenesis-Related Genes

**Figure 3:**
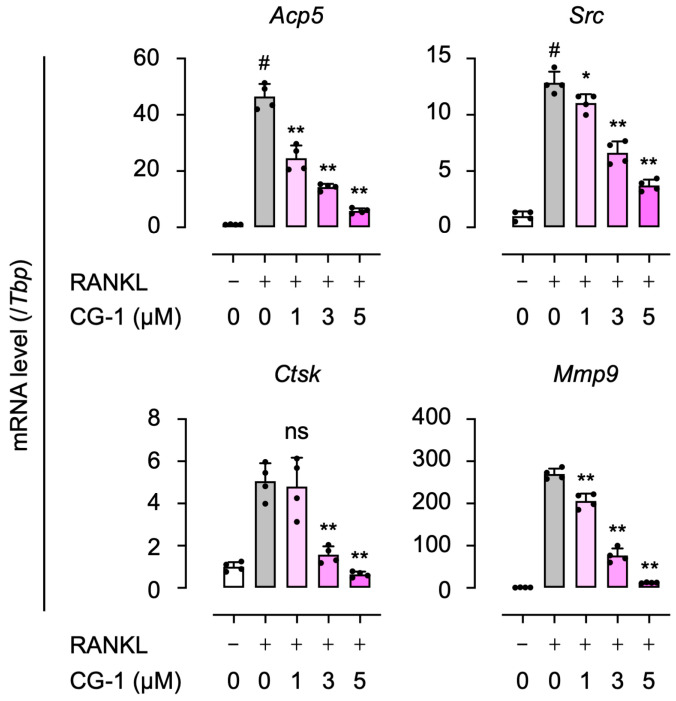
CG-1 attenuates the RANKL-induced expression of osteoclastogenesis-related genes. RAW264.7 cells were treated with GST-RANKL (50 ng/mL) and CG-1 (0–5 μM) for 5 days. Expression levels of the osteoclastogenesis-related genes were quantified by qRT-PCR. Data are presented as mean ± SD of four independent experiments. ns, not significant; # *p* < 0.01 vs. vehicle (0 μM); * *p* < 0.05 and ** *p* < 0.01 vs. GST-RANKL alone.

We examined the effects of CG-1 on the expression of the RANKL-induced osteoclastogenesis-related genes (*Acp5*, *Src*, *Ctsk*, and *Mmp9*) by qRT-PCR. Treatment with GST-RANKL increased the expression levels of all genes, *Acp5* (50.3 ± 8.2-fold), *Src* (18.8 ± 2.4-fold), *Ctsk* (5.2 ± 0.8-fold), and *Mmp9* (290 ± 45-fold), compared to those seen in GST alone-treated cells (Figure 3). The RANKL-induced gene expression was reduced in a concentration-dependent manner following co-treatment with CG-1 (1–5 μM). *Ctsk* expression tended to decrease with CG-1 treatment, though not significantly at lower concentrations (<5 μM). However, at 5 µM of CG-1, the expression levels of *Acp5*, *Src*, *Ctsk*, and *Mmp9* were suppressed by 79.6 ± 3.1%, 68.2 ± 4.3%, 78.3 ± 5.4%, and 88.7 ± 4.2% of the GST-RANKL alone-treated cells, respectively (Figure 3). These results indicate that CG-1 downregulated the osteoclastogenesis-related gene expression in RANKL-induced RAW264.7 cells.

### 3.5. CG-1 Suppresses RANKL-Induced NFATc1 Expression

**Figure 4:**
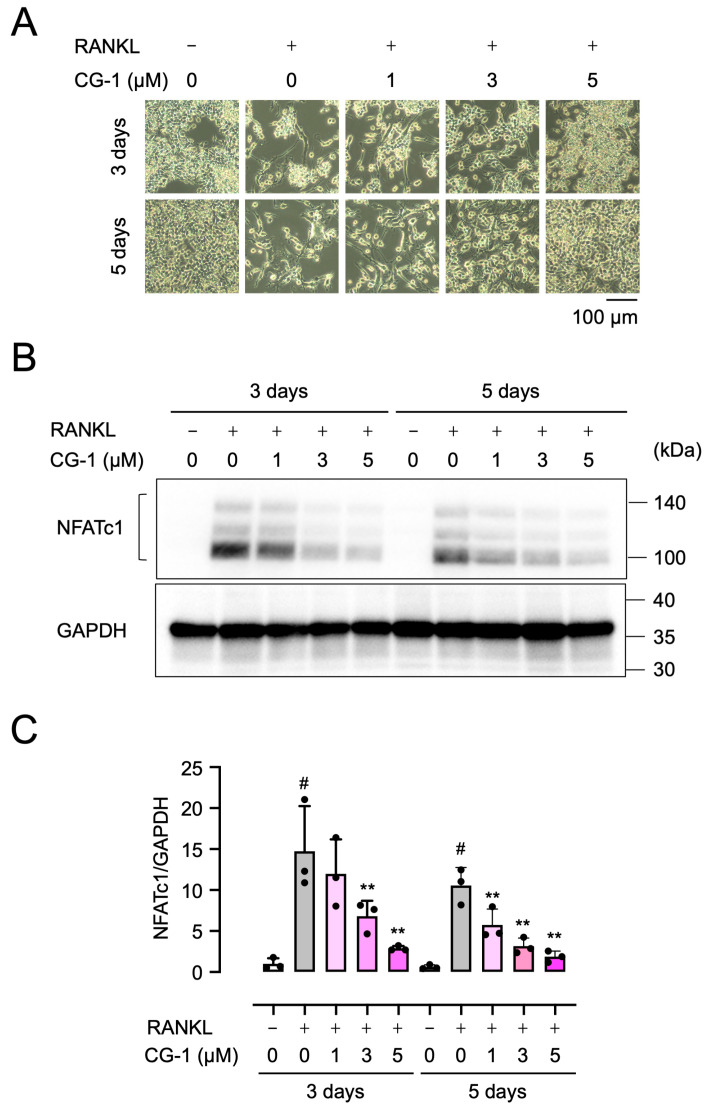
CG-1 suppresses RANKL-induced NFATc1 protein expression. (**A**) Suppression of RANKL-induced osteoclast differentiation by CG-1. RAW264.7 cells were treated with GST-RANKL (50 ng/mL) and CG-1 (0–5 µM) for 3 or 5 days. Representative images are shown (*n* = 3). (**B**) NFATc1 expression in RAW264.7 cells treated as stated in (**A**). Representative blots are shown (*n* = 3). (**C**) Densitometric quantification of the bands shown in (**B**). Data are presented as mean ± SD of three independent experiments. # *p* < 0.01 vs. vehicle (0 μM); ** *p* < 0.01 vs. RANKL alone.

To investigate the molecular mechanism underlying the suppression of RANKL-induced osteoclast differentiation by CG-1, we analyzed the time-dependent effects of RANKL and CG-1 during osteoclast differentiation of RAW264.7 cells. When the cells were incubated with GST-RANKL for 3 or 5 days, an extensive spread of cells, meaning the formation of osteoclast-like cells, was observed on day 3, and the large multinucleated cells, indicating the formation of mature osteoclasts, were detected on day 5. However, co-treatment with CG-1 (1–5 µM) suppressed these morphological changes in a concentration-dependent manner in RANKL-induced RAW 264.7 cells (Figure 4A).

Then we investigated the expression of NFATc1 by Western blot analysis (Figure 4B,C). GST-RANKL upregulated the expression of NFATc1 with the peak on day 3 (14.7 ± 5.5-fold) and then decreased on day 5 (10.6 ± 2.2-fold), compared to that seen in GST alone-treated cells. Moreover, when the cells were co-treated with CG-1 (1, 3, or 5 μM) in RANKL-induced cells, the NFATc1 expression was reduced by 18.7 ± 28.5%, 53.8 ± 12.8%, and 80.5 ± 2.1%, respectively, of RANKL alone-treated cells on day 3 (Figure 4C). On day 5, when the cells were treated with 1, 3, or 5 µM CG-1, NFATc1 expression decreased by 61.0 ± 13.0%, 78.5 ± 6.6%, and 87.4 ± 4.7%, respectively, of RANKL alone-treated cells. These results indicate that CG-1 suppressed NFATc1 expression in RANKL-induced RAW264.7 cells.

### 3.6. CG-1 Inhibits the Early Stage of Osteoclast Differentiation

**Figure 5:**
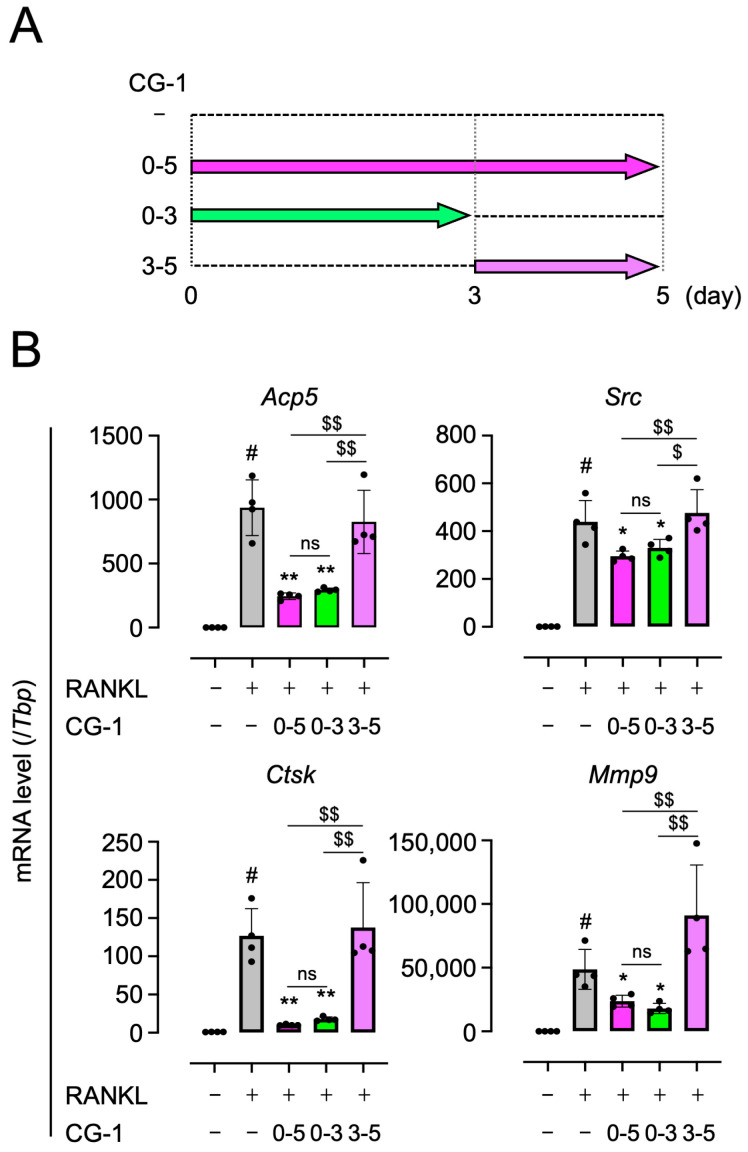
CG-1 suppresses the RANKL-induced osteoclastogenesis-related gene expression in the early stage of osteoclast differentiation. (**A**) Stage-specific effect of CG-1 on osteoclast differentiation in RANKL-induced RAW264.7 cells. CG-1 (5 µM) was added at the time points indicated by arrows. (**B**) Expression levels of osteoclastogenesis-related genes in RAW264.7 cells treated with GST-RANKL (50 ng/mL) and CG-1 (5 µM) for 5 days were determined by qRT-PCR. Data are presented as mean ± SD of four independent experiments. ns, not significant; # *p* < 0.01 vs. vehicle (0 μM); * *p* < 0.05 and ** *p* < 0.01 vs. GST-RANKL alone; $ *p* < 0.05 and $$ *p* < 0.01, between the indicated groups.

Since CG-1 suppressed the RANKL-induced NFATc1 expression on day 3 (Figure 4B,C), we further investigated the time-dependent effects of CG-1 on the suppression of osteoclast differentiation in RANKL-induced RAW264.7 cells. The cells were cultured in the presence of CG-1 during 0–5 days, 0–3 days, or 3–5 days of the 5-day osteoclast differentiation period (Figure 5A).

When the cells were treated with CG-1 on days 0–5, the RANKL-induced expression of *Acp5*, *Src*, *Ctsk*, and *Mmp9* was decreased by 73.9 ± 2.7%, 32.8 ± 5.1%, 92.0 ± 0.7%, and 51.3 ± 9.6%, respectively, of GST-RANKL alone-treated cells (Figure 5B). When the RANKL-induced cells were co-treated with CG-1 on days 0–3, the expression levels of these genes were almost similar to the results seen when CG-1 was added for 5 days. In contrast, when the cells were treated with CG-1 on days 3–5, the expression levels of these genes were almost the same as those in cells treated with RANKL alone (Figure 5B). In addition, there is no significant difference between the days 0–5 and days 0–3 treatment groups, whereas both groups differed significantly from the days 3–5 treatment group. These results indicate that CG-1 inhibited the early stage (0–3 days) of osteoclast differentiation in RANKL-induced RAW264.7 cells.

### 3.7. CG-1 Inhibits RANKL-Induced Phosphorylation of Akt

**Figure 6:**
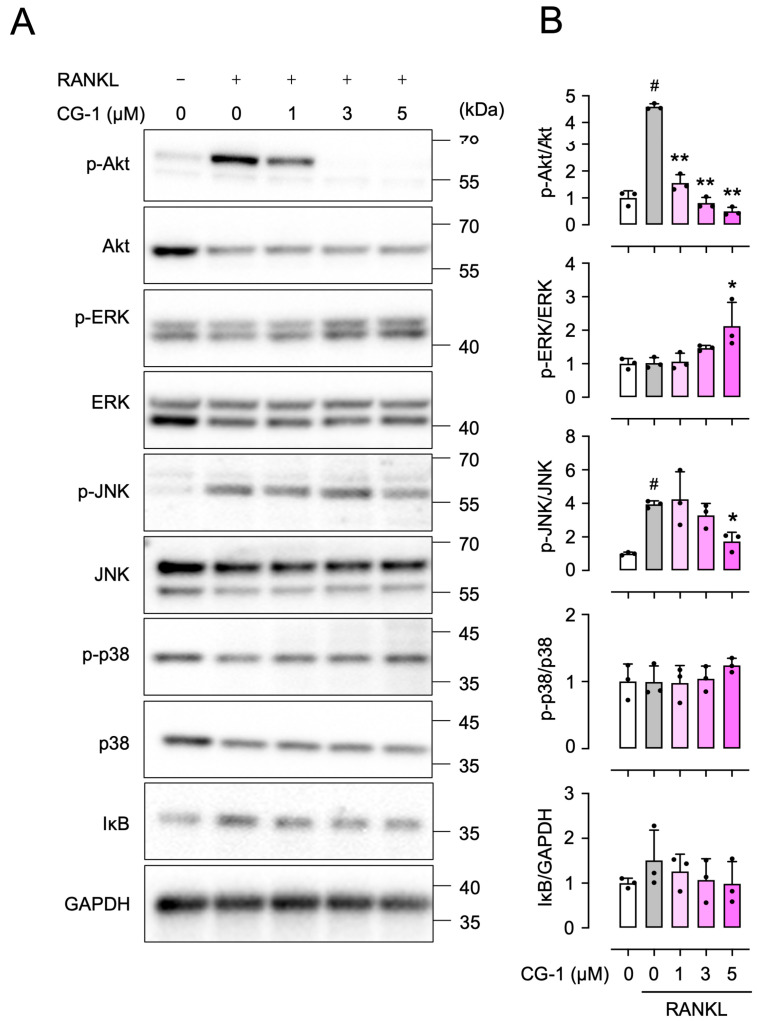
CG-1 inhibits RANKL-induced Akt phosphorylation. (**A**) Protein expression and phosphorylation in RAW264.7 cells treated with GST-RANKL (50 ng/mL) and CG-1 (0–5 µM) for 3 days. Representative blots are shown (*n* = 3). (**B**) Densitometric quantification of the bands shown in (**A**). Data are presented as the mean ± SD of three independent experiments. # *p* < 0.01 vs. vehicle (0 μM); * *p* < 0.05 and ** *p* < 0.01 vs. GST-RANKL alone.

To further elucidate the underlying molecular mechanism of CG-1-mediated suppression of RANKL-induced osteoclastogenesis, we investigated whether the PI3K/Akt, MAPK, and NF-κB signaling pathways are involved in this regulation. The PI3K/Akt and MAPK signaling pathways such as extracellular signal-regulated kinase (ERK), p38, and c-Jun N-terminal kinase (JNK) signaling pathways are involved in the regulation of osteoclast differentiation by controlling transcription factors such as NFATc1 [9]. In addition, the NF-κB signaling pathway is important in regulating osteoclastogenesis. Since its activation is associated with IκB degradation [23], we examined IκB degradation as an indicator of NF-κB activation. Treatment with RANKL increased Akt phosphorylation, although total Akt protein levels were decreased, compared to those in the untreated cells (Figure 6A,B). Moreover, co-treatment with GST-RANKL and CG-1 (1, 3, and 5 μM) reduced the Akt phosphorylation by 66.2 ± 6.7%, 82.4 ± 4.5%, and 89.1 ± 3.4%, respectively, of the GST-RANKL alone-treated cells (Figure 6A,B). Although the expression level of total Akt varied among the treatment groups, the phospho-Akt/GAPDH ratio (Supplementary Figure S3) was consistent with the p-Akt/Akt ratio (Figure 6B). ERK1/2 phosphorylation was not altered, even when RAW264.7 cells were treated with GST-RANKL (Figure 6A,B). In addition, up to 3 μM CG-1 showed no effect on ERK1/2 phosphorylation. However, when the cells were treated with 5 μM of CG-1, ERK1/2 phosphorylation was increased by 108 ± 69.0% of GST-RANKL alone-treated ones (Figure 6A,B). Furthermore, JNK phosphorylation was elevated by treatment with GST-RANKL at 3.9 ± 0.2-fold, compared to that seen in the untreated cells (Figure 6A,B). Its enhancement was reduced by 56 ± 14.0% when the cells were co-treated with 5 μM of CG-1 (Figure 6A,B). In contrast, p38 phosphorylation (p-p38) and IκB levels were not markedly altered by either GST-RANKL or CG-1 treatment (Figure 6A,B). These findings suggest that CG-1 inhibited the PI3K/Akt signaling pathway in RANKL-induced RAW264.7 cells. In addition, high concentrations of CG-1 (5 μM) suppressed the JNK signaling pathway.

### 3.8. Pharmacological Inhibition of the PI3K/Akt Signaling Pathway in RANKL-Induced RAW264.7 Cells

**Figure 7:**
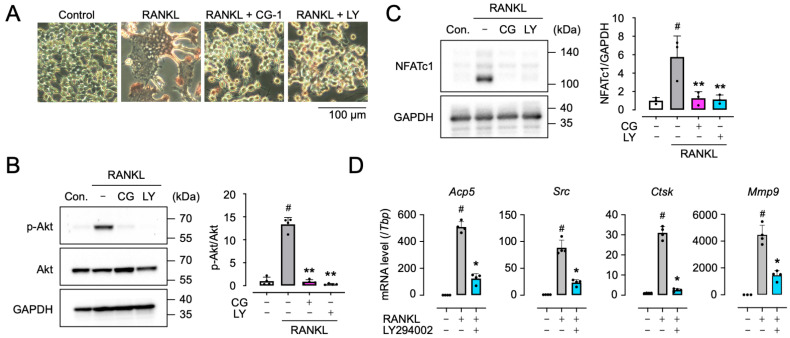
PI3K/Akt inhibitor LY294002 represses RANKL-induced osteoclastogenesis. (**A**) TRAP staining of RAW264.7 cells treated with GST-RANKL (50 ng/mL) in the presence of CG-1 (5 µM) or LY294002 (LY, 5 µM) for 5 days. Representative images are shown (*n* = 3). (**B**) Western blot analysis of Akt and phospho-Akt (p-Akt). The cells were treated for 3 days as stated in (**A**) (left). Band intensity was quantified and the p-Akt/Akt ratio is shown (right) (*n* = 4). Con., control; CG, CG-1; # *p* < 0.01 vs. vehicle treatment (0 μM); ** *p* < 0.01 vs. GST-RANKL alone. (**C**) Western blot analysis of NFATc1 (left). The cells were cultured for 3 days as stated in (**B**). Band intensity was quantified (right) (*n* = 3). # *p* < 0.01 vs. vehicle treatment (0 μM); ** *p* < 0.01 vs. GST-RANKL alone (**D**). qRT-PCR analysis of gene expression in cells treated as described in (**A**). Data are presented as the mean ± SD of 3–4 independent experiments. # *p* < 0.01 vs. vehicle treatment (0 μM); * *p* < 0.05 vs. GST-RANKL alone.

To confirm the involvement of the PI3K/Akt signaling pathway in CG-1-suppressed RANKL-induced osteoclast differentiation, RAW264.7 cells were treated with GST-RANKL together with CG-1 or LY294002 (the PI3K inhibitor). When GST-RANKL-treated RAW264.7 cells were co-treated with CG-1 or LY294002, the number of RANKL-induced TRAP-positive multinucleated cells was decreased by each treatment (Figure 7A). Moreover, LY294002 inhibited RANKL-induced Akt phosphorylation by 97.6 ± 1.2% of GST-RANKL alone-treated cells, which was comparable to CG-1-mediated suppression (92.4 ± 3.3%) (Figure 7B). In addition, LY294002 reduced the RANKL-induced NFATc1 expression by 80.3 ± 8.5% of GST-RANKL alone-treated cells, whose results were similar to CG-1 treatment (77.9 ± 12.4%) (Figure 7C). Furthermore, LY294002 downregulated the expression of osteoclastogenesis-related genes, *Acp5*, *Src*, *Ctsk*, and *Mmp9*, by 75.6 ± 7.3%, 73.5 ± 6.4%, 91.8 ± 2.2%, and 67.7 ± 8.1%, respectively, of RANKL alone-treated cells (Figure 7D), which are similar to the results obtained when CG-1 was used (Figure 3). These results indicate that CG-1 inhibited osteoclast differentiation by inhibiting the PI3K/Akt signaling pathway in RANKL-induced RAW264.7 cells.

## 4. Discussion

Osteoporosis is a metabolic bone disease that is characterized by low bone mass and degradation of bone microstructure, and is seen more frequently in older individuals, especially postmenopausal women [28]. Inhibition of osteoclast function may provide a novel therapeutic strategy for treating osteoclast-related diseases. Several anti-resorptive drugs have been used in clinical settings. Bisphosphonates are the most utilized anti-resorptive drugs, but have some adverse effects such as osteonecrosis of the jaw and atypical femoral fractures following prolonged administration [29,30]. Natural compounds with the ability to support bone health are interesting substances because they have milder effects and fewer adverse effects than those seen with synthetic molecules [31].

**Figure 8:**
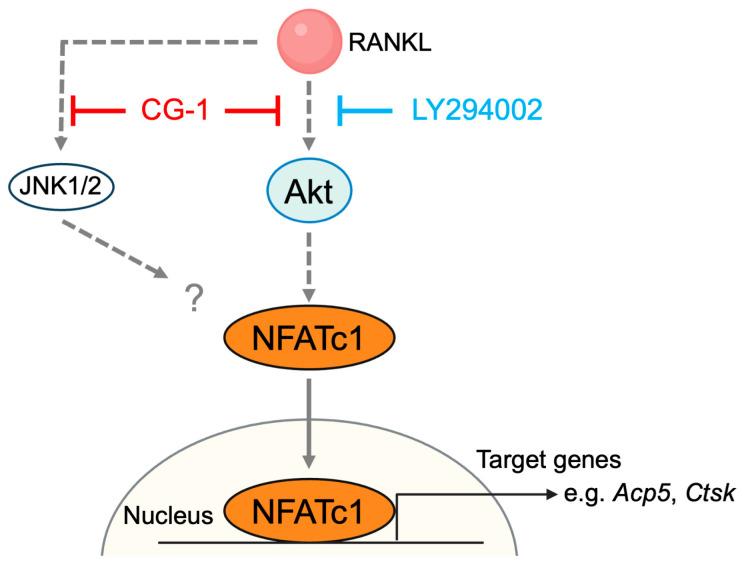
Proposed molecular mechanism involved in the anti-osteoclastogenic effect of CG-1 in RANKL-induced RAW264.7 cells.

Plants are a rich source of bioactive compounds that have a variety of biological activities. Natural compounds, including polyphenols, alkaloids, flavonoids, and limonoids, found in plants have multifaceted properties, such as anti-inflammatory, antioxidant, and cell-regulatory activities [32,33,34]. Previous studies have shown that some plant-derived compounds improve bone health [35,36]. Andiroba [*Carapa guianensis* (Meliaceae)] is a traditional tropical tree in South America [37,38]. Andiroba oil extracted from its seeds has various biological activities such as decreasing inflammation and increasing wound healing [39]. In addition, andiroba oil contains various limonoids, including CG-1 (7-deacetoxy-7-oxogedunin), which has been reported to improve insulin resistance and suppress lipid accumulation [20,21,22]. In this study, we identified a novel property of a limonoid: CG-1 is a bioactive limonoid with the potential to act as an anti-resorptive agent and promote bone health. CG-1 attenuated RANKL-induced osteoclastogenesis in RAW264.7 cells via inhibiting the PI3K/Akt-NFATc1 axis in RAW264.7 cells (Figure 8). Various plant-derived compounds have been reported to attenuate RANKL-induced osteoclast differentiation by suppressing the phosphorylation of Akt and MAPKs (ERK, JNK, and p38) [18,40,41,42]. However, we have not identified the direct target of CG-1 in the suppression of osteoclast differentiation. Akt or one of its upstream molecules may be the candidate for CG-1’s target. Further study is needed to confirm this.

RANKL and its receptor, RANK, are key regulators of bone remodeling and regulate bone resorption via several intracellular signaling cascades, including the MAPK and PI3K/Akt signaling pathways [43]. Both the MAPK and PI3K/Akt signaling pathways are critical for the regulation of osteoclast differentiation; however, Akt also plays a crucial role in promoting osteoclast survival [44]. CG-1 suppressed Akt phosphorylation, and these results are almost the same as those when the PI3K inhibitor, LY294002 was used, suggesting that CG-1 suppressed osteoclast differentiation by inhibiting the PI3K/Akt signaling pathway in RANKL-induced RAW264.7 cells (Figure 6). Interestingly, RANKL treatment decreased total Akt protein levels (Figure 6). This may reflect the remodeling of intracellular signaling pathways and the change in the turnover of Akt protein. In contrast, the suppressive effect of CG-1 on JNK activation was limited and ERK phosphorylation was slightly elevated (Figure 6). In general, activation of the ERK signaling pathway promotes osteoclast differentiation [45]. However, ERK1/2 phosphorylation increased with 5 μM of CG-1 (Figure 6). These results may suggest that ERK was activated as a compensatory response to the inhibition of the PI3K/Akt signaling pathway, and osteoclast differentiation was not completely inhibited by 5 µM CG-1. Furthermore, reactive oxygen species (ROS) play an important role in promoting the signaling pathways that regulate osteoclast differentiation [46,47]. Several natural compounds, including 4-methylcatechol, oroxylin A, notopterol, tussilagone, and alpinetin, have been reported to suppress ROS production and inhibit osteoclast differentiation by modulating the Keap1/Nrf2 pathway [48,49,50,51,52]. Since it has been reported that CG-1 has antioxidant properties [53], this pathway may be involved in CG-1-suppressed osteoclast differentiation. Thus, future studies are needed to clarify this possibility. NFATc1 is the master transcription factor for osteoclastogenesis, which regulates the expression of the osteoclastogenesis-related genes such as *Acp5*, *Src*, *Ctsk*, and *Mmp9* by binding directly to their promoter regions [54]. NFATc1 expression is elevated by activating the PI3K/Akt signaling pathway during RANKL-induced osteoclastogenesis [9]. In this study, we found that CG-1 attenuated the early stage of osteoclastogenesis by reducing NFATc1 expression through suppressing the PI3K/Akt signaling pathway in RANKL-induced RAW264.7 cells. Previous study showed that 7-oxo-7-deacetoxygedunin, i.e., CG-1, inhibits osteoclastogenesis by suppressing the NF-κB and MAPK signaling pathways in RANKL-induced RAW264.7 cells [23]. In the present study, the involvement of the MAPK and NF-κB signaling pathways in this regulation was limited (Figure 6). These differences may be due to the time point of the analysis. The previous study examined at 60 min (very early stage of osteoclastogenesis) after RANKL stimulation. In contrast, we examined at 3 days after RANKL stimulation and found that the NFATc1 and the osteoclast-related genes were abundantly expressed. Thus, this difference may reflect not merely methodological variation, but rather a biologically distinct stage of osteoclast differentiation. Suppression of Akt phosphorylation and NFATc1 expression by CG-1, as well as its stage-specific effects during osteoclastogenesis, reveal that CG-1 is involved in regulating the initiation stage (very early stage at 60 min) and the progression stage (early stage, days 0–3), but not the maturation stage (late stage, days 3–5), of osteoclast differentiation. This should be further elucidated. Additionally, the present study has limitations. All experiments were performed using RAW264.7 cells, which do not fully reflect the physiological properties of primary osteoclast precursors. The anti-osteoclastogenic effects of CG-1 should be confirmed using the primary macrophages such as bone marrow-derived macrophages and animal models to understand the whole regulatory mechanism of CG-1-mediated inhibition of osteoclastogenesis and bone health.

## 5. Conclusions

We showed that andiroba limonoid CG-1 attenuated the early stage of RANKL-induced osteoclast differentiation by inhibiting the PI3K/Akt–NFATc1 axis.

## Data Availability

The original contributions presented in this study are included in the article/Supplementary Materials. Further inquiries can be directed to the corresponding author.

## Supplementary Materials

The following supporting information can be downloaded at: https://www.mdpi.com/article/10.3390/cells15100854/s1. Method S1: Preparation of glutathione S-transferase (GST)-RANKL expression vector; Method S2: Expression and purification of recombinant GST-RANKL; Figure S1: Expression of the recombinant GST-RANKL and GST proteins. Purified GST-RANKL and GST proteins were resolved by SDS-PAGE and visualized by CBB staining. The expected molecular weights of GST-RANKL and GST are approximately 45 and 26 kDa, respectively. M, molecular weight marker; Figure S2: TRAP staining. RAW264.7 cells were cultured with sRANKL (50 ng/mL), GST-RANKL (50 ng/mL), or GST (50 ng/mL) for 5 days. TRAP-positive cells were counted in three randomly selected fields per experiment. The experiments were independently repeated three times. Representative images are shown; Figure S3: p-Akt/GAPDH ratio in CG-1-treated RANKL-induced RAW264.7 cells. The cells were treated with GST-RANKL (50 ng/mL) and CG-1 (0–5 μM) for 3 days. p-Akt level was normalized to GAPDH level (*n* = 3). # *p* < 0.01 vs. vehicle treatment (0 μM); ** *p* < 0.01 vs. GST-RANKL alone. Reference [25] is cited in the Supplementary Materials.

## Abbreviations

The following abbreviations are used in this manuscript:

M-CSF	macrophage colony-stimulating factor
NF-κB	nuclear factor-κB
RANKL	receptor activator of nuclear factor-κB (NF-κB) ligand
PI3K	phosphoinositide 3-kinase
Akt	protein kinase B
NFATc1	nuclear factor of activated T-cells cytoplasmic 1
Acp5	acid phosphatase 5
Trap	tartrate-resistant acid phosphatase
Src	proto-oncogene tyrosine-protein kinase Src
Ctsk	cathepsin K
Mmp9	matrix metallopeptidase 9
MAPK	mitogen-activated protein kinase
DMSO	dimethyl sulfoxide
sRANKL	soluble RANKL
GST	glutathione S-transferase
DMEM	Dulbecco’s modified eagle’s medium
FCS	fetal calf serum
PS	Penicillin–streptomycin mixed solution
MEM	Minimum essential medium
NEAA	MEM non-essential amino acids solution
qRT-PCR	quantitative real-time PCR
Tbp	TATA-box binding protein
HRP	horseradish peroxidase
GAPDH	glyceraldehyde-3-phosphate dehydrogenase
SD	standard deviation
ANOVA	analysis of variance
ERK	extracellular signal-regulated kinase
JNK	c-Jun N-terminal kinase
ROS	reactive oxygen species
