# Supplementary material for: Limonoid 7-Deacetoxy-7-oxogedunin (CG-1) Attenuates RANKL-Induced Osteoclastogenesis via Inhibiting PI3K/Akt-NFATc1 Axis
Source: Cells. 2026 May 7;15(10):854. doi: 10.3390/cells15100854 (PMC13204205; doi:10.3390/cells15100854)
Supplement: Supplementary file 1 [file cells-15-00854-s001.zip › cells-4198705-supplementary.pdf]

Supporting Information

Supplemental Methods

Method S1. Preparation of glutathione S-transferase (GST)-RANKL expression vector

Total RNA was extracted by RNAiso Plus (Takara-Bio) according to the manufacturer's protocols. Mouse MC3T3-E1 cells (RIKEN BioResource Research Center; Ibaraki, Japan) were differentiated into osteoblasts for 5 days in α-MEM (Sigma-Aldrich, St. Louis, MO, USA) supplemented with 10% (v/v) fetal calf serum, 50 μg/mL ascorbic acid (Nacalai Tesque, Kyoto, Japan), and a solution of 50 U/mL penicillin and 50 μg/mL streptomycin (Nacalai Tesque). First-strand cDNAs were synthesized from total RNA by reverse transcription. Using the cDNAs as the templates, the DNA region (468 bp) carrying the extracellular domain of RANKL (amino acid residues 157–316) [25] was amplified by PCR with the specific primers: 5'-ATT<u>GTCGAC</u>GAGGCAAGCCTG-AGGCCCA-5' and 5'-AGA<u>GCGGCCGC</u>TCAGTCTATGTCCTGAACTTTG-3' with *Sal*I and *Not*I restriction enzyme recognition sites (*underlined*), respectively. The resultant PCR products (RANKL extracellular domain) were ligated into the pGEX-6P-1 vector (Cytiva; Marlborough, MA, USA) to make pGEX-6P-1-RANKL vector to express the recombinant RANKL extracellular domain as an N-terminal GST fusion protein.

Method S2. Expression and purification of recombinant GST-RANKL

The pGEX-6P-1-RANKL or its empty vector pGEX-6P-1 was transformed into *Escherichia coli* BL21 (DE3) (NIPPON GENE, Tokyo, Japan). The transformant carrying pGEX-6P-1-RANKL or pGEX-6P-1 was cultured in LB broth at 30 °C on the orbital shaker. When the optical density at 600 nm (OD_{600}) reached 0.6–0.8, isopropyl-β-D-thiogalactopyranoside (Nacalai Tesque) was added to a final concentration of 0.1 mM, and the culture was continued for an additional 5 h at 30 °C to produce the recombinant GST-RANKL or GST.

The cells were harvested by centrifugation and washed with PBS(-). The cell pellets were resuspended in GST buffer [20 mM Tris-HCl (pH 8.0), 1 mM EDTA, 1% (v/v) Triton X-100, 5 mM DTT, and a protease inhibitor cocktail (Nacalai Tesque)] and disrupted by sonication, followed by clarified by centrifugation to remove cell debris. The resultant supernatant containing the soluble GST-RANKL or GST protein was mixed with 500 μL of Glutathione-Sepharose 4B beads (Cytiva) that were pre-equilibrated with GST buffer containing 1 mM DTT. The mixture was incubated overnight at 4 °C with gentle agitation. The beads-lysate mixture was then loaded onto the column and washed with GST buffer. The GST-RANKL or GST protein bound with beads was subsequently eluted with High Salt Elution Buffer [100 mM Tris-HCl (pH 8.0), 120 mM NaCl, 20 mM reduced glutathione]. The eluted fractions were concentrated and the buffer was exchanged to PBS(-) containing 10% (v/v) glycerol using a Pierce Protein Concentrator (PES, 30K MWCO; Thermo Fisher Scientific, Waltham, MA, USA). The protein concentration was determined by a BCA Protein Assay Kit (Nacalai Tesque). The purity of each protein was assessed by SDS-PAGE using a 5–20% gradient gel (ATTO, Tokyo, Japan), followed by Coomassie Brilliant Blue (CBB) staining.

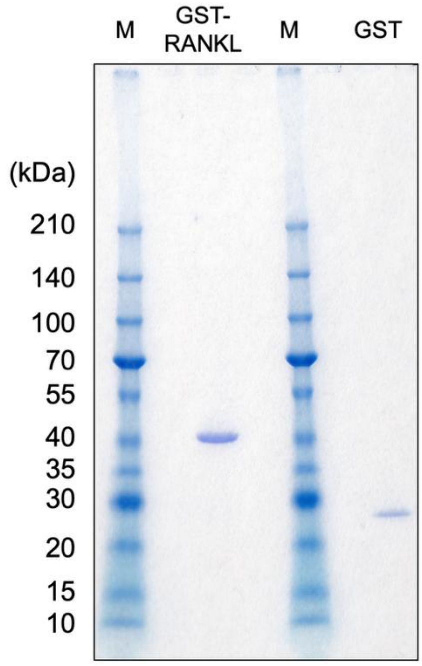

Figure S1. Expression of the recombinant GST-RANKL and GST proteins. Purified GST-RANKL and GST proteins were resolved by SDS-PAGE and visualized by CBB staining. The expected molecular weights of GST-RANKL and GST are approximately 45 and 26 kDa, respectively. M, molecular weight marker.

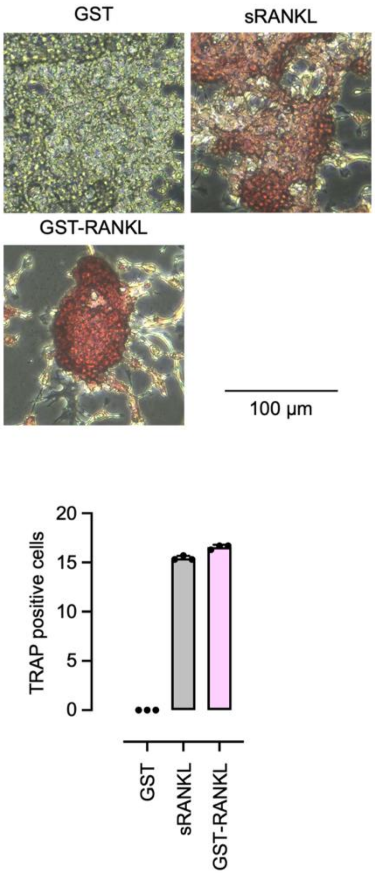

Figure S2. TRAP staining. RAW264.7 cells were cultured with sRANKL (50 ng/mL), GST-RANKL (50 ng/mL), or GST (50 ng/mL) for 5 days. TRAP-positive cells were counted in three randomly selected fields per experiment. The experiments were independently repeated three times. Representative images are shown.

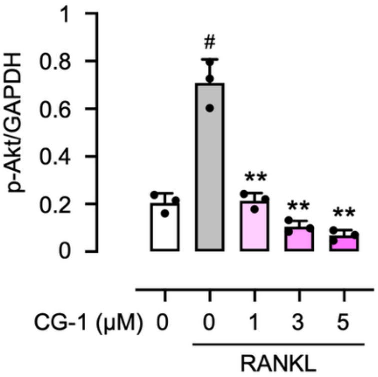

Figure S3. p-Akt/GAPDH ratio in CG-1-treated RANKL-induced RAW264.7 cells. The cells were treated with GST-RANKL (50 ng/mL) and CG-1 (0–5 μM) for 3 days. p-Akt level was normalized to GAPDH level (n = 3). #$p < 0.01$ vs. vehicle treatment (0 μM); **$p < 0.01$ vs. GST-RANKL alone.
